# Acridine form IX

**DOI:** 10.1107/S2056989019003645

**Published:** 2019-03-26

**Authors:** Peter W. Stephens, Einat Schur, Saul H. Lapidus, Joel Bernstein

**Affiliations:** a Department of Physics and Astronomy, Stony Brook, NY 11794-3800, USA; b Department of Chemistry, Ben Gurion University of the Negev, Beer Sheva, 84105, Israel; c X-ray Science Division, Argonne National Laboratory, Lemont, IL 60439, USA

**Keywords:** crystal structure, powder diffraction, acridine, polymorph

## Abstract

We report a new polymorph of acridine, C_13_H_9_N, denoted form IX, obtained as thin needles by slow evaporation of a toluene solution. The structure was solved and refined from powder X-ray data. The structures of five unsolvated forms were previously known, but this is only the second with one mol­ecule in the asymmetric unit. The melting point [differential scanning calorimetry (DSC) onset] and heat of fusion are 108.8 (3) °C and 19.2 (4) kJ mol^−1^, respectively.

## Abstract 2

A new polymorph of acridine was obtained during a study of the polymorphism of that mol­ecule. This structure was previously predicted in a computational search.

## Chemical context   

With the crystal structures of five forms already reported, acridine is already one of the more prolifically polymorphic mol­ecules known [see Phillips (1956 ▸), Phillips *et al.* (1960 ▸), Mei & Wolf (2004 ▸), Braga *et al.* (2010 ▸), Kupka *et al.* (2012 ▸), and Lusi *et al.* (2015 ▸)]; two additional forms have been described, but structures were not reported, by Herbstein & Schmidt (1955 ▸) and Braga *et al.* (2010 ▸). This large number of observed forms seems particularly noteworthy in view of the fact that the mol­ecule has zero degrees of flexibility, although perhaps counterintuitively, some 40 rigid mol­ecules are observed to be polymorphic (Cruz-Cabeza & Bernstein, 2013 ▸).
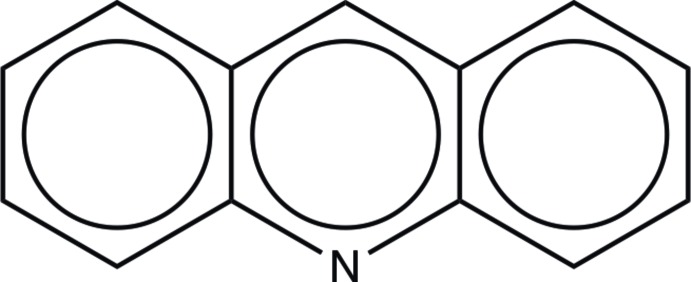


## Structural commentary   

The form described here was previously predicted by Price & Price (unpublished) using *CrystalPredictor* (Karamertzanis & Panti­lides, 2005 ▸) to generate a crystal energy landscape, limited to one independent mol­ecule in the asymmetric unit cell in the most common space groups. These were relaxed to mechanically stable structures with *DMACRYS* (Price *et al.*, 2010 ▸). This new form corresponded to one of two structures with the lowest computed lattice energy. Further details are available in Schur *et al.* (2019 ▸). Geometry details for form IX are given in Table 1 ▸.

## Supra­molecular features   

The four mol­ecules in the unit cell are connected by a cycle of C⋯H (2.81 Å) and N⋯H (2.73 Å) contacts that are shorter than the sum of the van der Waals radii. There is also an H⋯H inter­action of 2.29 Å.

## Synthesis and crystallization   

Crystals were grown by slow evaporation from a toluene solution. Thin needles of form IX samples were taken from the walls of crystallization vials. The material was gently crushed and loaded into a glass capillary for powder diffraction measurements. Further details are available in Schur (2013 ▸).

## Refinement details   

Crystal data, data collection and structure refinement details are summarized in Table 2 ▸. Data were collected at the high resolution powder diffractometer at the National Synchrotron Light Source beamline X16C, operated in step scanning mode. X-rays of wavelength 0.69979 Å were selected by a Si(111) channel cut monochromator. Diffracted X-rays were selected by a Ge(111) analyzer before an NaI(Tl) scintillation detector. The sample of form IX was obtained concomitantly with forms III (1.4%) and VII (1.1%), which were included in the Rietveld fit, with atomic positions fixed at literature values.

The mol­ecule was defined by a *z*-matrix for refinement. Mirror symmetry was imposed on bond distances and angles; 7 distances, 6 angles, and 11 torsions were refined. There is a single isotropic displacement parameter for all C and N atoms; that of H atoms is 1.5 times greater. All H atoms are tethered.

Standard uncertainties were calculated by a bootstrap method, described in Coelho (2016 ▸). As such, they reflect the propagation of statistical errors from the raw data and do not take account of systematic errors. Realistic estimates of the precision of measurements are somewhat larger.

**Figure 1:**
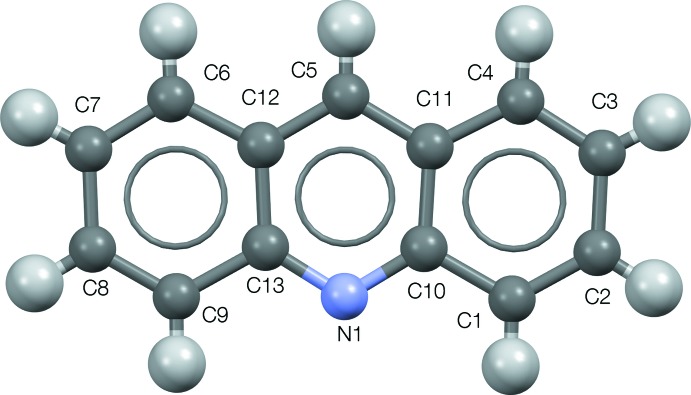
The acridine mol­ecule in form IX, with atom labels and 50% probability displacement spheres.

**Figure 2:**
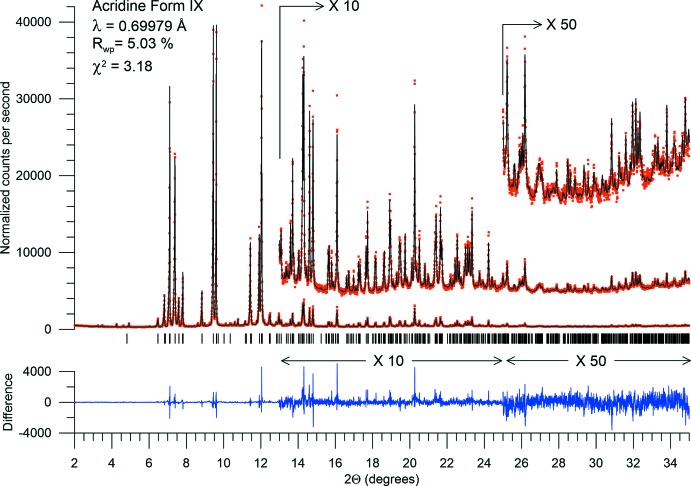
Rietveld plot of acridine form IX. Red dots are measured intensities, black line is the fit, and the blue trace at the bottom is the difference plot, measured minus fit. Note the two vertical scale changes. Vertical tick lines show allowed peak positions of form IX peaks. Fit includes two impurity phases: 1.4% form III and 1.1% form VII. Tick marks were omitted for clarity.

**Figure 3:**
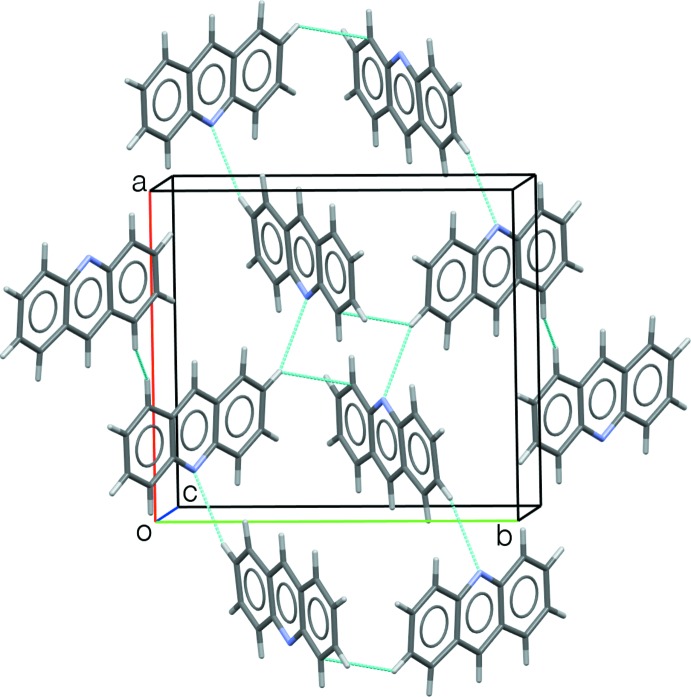
Packing diagram of acridine form IX. Close inter­molecular inter­actions (less than the sum of van der Waals radii) are marked in turquoise dashed lines.

The Rietveld refinement plot is shown in Fig. 1 ▸. Fig. 2 ▸ illustrates the atom-labeling scheme, and Fig. 3 ▸ shows the three-dimensional structure, with short inter­molecular inter­actions shown as broken lines.

The refinement model included preferred orientation parameter 1.08 in the (100) direction (March, 1932 ▸; Dollase, 1986 ▸), and anisotropic microstrain broadening (Stephens, 1999 ▸).

## Supplementary Material

Crystal structure: contains datablock(s) global, I. DOI: 10.1107/S2056989019003645/eb2017sup1.cif

Rietveld powder data: contains datablock(s) I. DOI: 10.1107/S2056989019003645/eb2017Isup2.rtv

Supporting information file. DOI: 10.1107/S2056989019003645/eb2017Isup3.cml

CCDC reference: 1869547

Additional supporting information:  crystallographic information; 3D view; checkCIF report

## Figures and Tables

**Table 1:** Selected geometric parameters (Å, °)

N1—C10	1.315 (18)	C5—C12	1.388 (6)
N1—C13	1.317 (18)	C6—C7	1.366 (17)
C1—C2	1.37 (3)	C6—C12	1.436 (12)
C1—C10	1.44 (3)	C7—C8	1.41 (3)
C2—C3	1.41 (2)	C8—C9	1.36 (4)
C3—C4	1.367 (16)	C9—C13	1.44 (4)
C4—C11	1.435 (9)	C10—C11	1.444 (7)
C5—C11	1.389 (5)	C12—C13	1.443 (11)
			
C10—N1—C13	116.9 (7)	N1—C10—C11	124.5 (8)
C2—C1—C10	121.3 (12)	C1—C10—C11	116.6 (11)
C1—C2—C3	121.7 (17)	C4—C11—C5	122.5 (5)
C2—C3—C4	119.7 (13)	C4—C11—C10	120.0 (7)
C3—C4—C11	120.8 (10)	C5—C11—C10	117.6 (5)
C11—C5—C12	118.9 (4)	C5—C12—C6	122.4 (8)
C7—C6—C12	120.9 (14)	C5—C12—C13	117.7 (6)
C6—C7—C8	119.4 (15)	C6—C12—C13	119.8 (8)
C7—C8—C9	122 (2)	N1—C13—C9	119.0 (13)
C8—C9—C13	121.3 (18)	N1—C13—C12	124.5 (9)
N1—C10—C1	118.9 (10)	C9—C13—C12	116.5 (13)

**Table 2:** Experimental details

Crystal data	
Chemical formula	C_13_H_9_N
*M*_r_	179.21
Crystal system, space group	Monoclinic, *P*2_1_/*n*
Temperature (K)	295
*a*, *b*, *c* (Å)	11.28453 (11), 12.38182 (12), 6.67905 (9)
β (°)	92.0618 (6)
*V* (Å^3^)	932.61 (2)
*Z*	4
Radiation type	Synchrotron, λ = 0.699789 Å
μ (mm^−1^)	0.08
Specimen shape, size (mm)	Cylinder, 8 × 1

Data collection	
Diffractometer	Huber 401 diffractometer, Ge(111) analyzer crystal
Specimen mounting	1 mm glass capillary, spun during data collection
Data collection mode	Transmission
Scan method	Step
2θ values (°)	2θ_min_ = 2, 2θ_max_ = 35, 2θ_step_ = 0.005

Refinement	
*R* factors and goodness of fit	*R*_p_ = 0.041, *R*_wp_ = 0.050, *R*_exp_ = 0.028, *R*_Bragg_ = 0.011, χ^2^ = 3.183
No. of parameters	81
No. of restraints	12
H-atom treatment	H-atom parameters not refined

Computer programs: *TOPAS-Academic* (Coelho, 2016 ▸) and *Mercury* (Macrae *et al.*, 2008 ▸).

## supplementary crystallographic information

### Crystal data

**Table:**

C_13_H_9_N	*Z* = 4
*M**_r_* = 179.21	*D*_x_ = 1.276 Mg m^−^^3^
Monoclinic, *P*2_1_/*n*	Synchrotron radiation, λ = 0.699789 Å
*a* = 11.28453 (11) Å	µ = 0.08 mm^−^^1^
*b* = 12.38182 (12) Å	*T* = 295 K
*c* = 6.67905 (9) Å	Particle morphology: thin needles
β = 92.0618 (6)°	yellow-white
*V* = 932.61 (2) Å^3^	cylinder, 8 × 1 mm

### Data collection

**Table:**

Huber 401 diffractometer, Ge(111) analyzer crystal	Data collection mode: transmission
Radiation source: National Synchrotron Light Source	Scan method: step
Channel cut Si(111) monochromator	2θ_min_ = 2°, 2θ_max_ = 35°, 2θ_step_ = 0.005°
Specimen mounting: 1 mm glass capillary, spun during data collection	

### Refinement

**Table:**

Least-squares matrix: full	12 restraints
*R*_p_ = 0.041	22 constraints
*R*_wp_ = 0.050	H-atom parameters not refined
*R*_exp_ = 0.028	Weighting scheme based on measured s.u.'s
*R*_Bragg_ = 0.011	(Δ/σ)_max_ = 0.02
6601 data points	Background function: 9th order Chebyshev plus broad pseudo-Voigt
Profile function: Convolution of Gaussian and Lorentzian, with anisotropic strain broadening per Stephens (1999).	Preferred orientation correction: March parameter 1.084 in (1 0 0) direction
81 parameters	

### Special details

**Table:**

Refinement. Mirror symmetry imposed on bond distances and angles.

### Fractional atomic coordinates and isotropic or equivalent isotropic displacement parameters (Å^2^)

**Table:**

	*x*	*y*	*z*	*U*_iso_*/*U*_eq_	
N1	0.1540 (5)	0.1053 (19)	0.045 (2)	0.0474 (5)*	
C1	0.1213 (11)	0.003 (3)	−0.252 (4)	0.0474 (5)*	
C2	0.1640 (14)	−0.048 (2)	−0.417 (3)	0.0474 (5)*	
C3	0.2853 (15)	−0.0476 (11)	−0.4573 (15)	0.0474 (5)*	
C4	0.3633 (9)	0.0068 (7)	−0.3327 (10)	0.0474 (5)*	
C5	0.4001 (2)	0.1154 (2)	−0.0256 (3)	0.0474 (5)*	
C6	0.4263 (13)	0.2290 (7)	0.2776 (12)	0.0474 (5)*	
C7	0.379 (2)	0.2748 (11)	0.4427 (15)	0.0474 (5)*	
C8	0.256 (2)	0.266 (2)	0.471 (3)	0.0474 (5)*	
C9	0.1830 (18)	0.210 (4)	0.341 (6)	0.0474 (5)*	
C10	0.1992 (5)	0.0588 (10)	−0.1120 (15)	0.0474 (5)*	
C11	0.3234 (4)	0.0603 (4)	−0.1568 (6)	0.0474 (5)*	
C12	0.3543 (7)	0.1655 (3)	0.1406 (7)	0.0474 (5)*	
C13	0.2282 (7)	0.1578 (9)	0.1666 (17)	0.0474 (5)*	
H1	0.0388 (12)	0.002 (4)	−0.229 (6)	0.0711 (8)*	
H2	0.1103 (18)	−0.086 (2)	−0.504 (4)	0.0711 (8)*	
H3	0.313 (2)	−0.0838 (17)	−0.572 (2)	0.0711 (8)*	
H4	0.4453 (10)	0.0071 (13)	−0.3603 (18)	0.0711 (8)*	
H5	0.4825 (3)	0.1185 (6)	−0.0491 (8)	0.0711 (8)*	
H6	0.5086 (12)	0.2370 (14)	0.256 (2)	0.0711 (8)*	
H7	0.428 (3)	0.315 (2)	0.535 (3)	0.0711 (8)*	
H8	0.224 (3)	0.299 (3)	0.586 (4)	0.0711 (8)*	
H9	0.1006 (19)	0.205 (6)	0.364 (7)	0.0711 (8)*	

### Geometric parameters (Å, º)

**Table:**

N1—C10	1.315 (18)	C9—C13	1.44 (4)
N1—C13	1.317 (18)	C10—C11	1.444 (7)
C1—C2	1.37 (3)	C12—C13	1.443 (11)
C1—C10	1.44 (3)	C1—H1	0.95
C2—C3	1.41 (2)	C2—H2	0.95
C3—C4	1.367 (16)	C3—H3	0.95
C4—C11	1.435 (9)	C4—H4	0.95
C5—C11	1.389 (5)	C5—H5	0.95
C5—C12	1.388 (6)	C6—H6	0.95
C6—C7	1.366 (17)	C7—H7	0.95
C6—C12	1.436 (12)	C8—H8	0.95
C7—C8	1.41 (3)	C9—H9	0.95
C8—C9	1.36 (4)		
			
C10—N1—C13	116.9 (7)	N1—C13—C12	124.5 (9)
C2—C1—C10	121.3 (12)	C9—C13—C12	116.5 (13)
C1—C2—C3	121.7 (17)	C2—C1—H1	120 (4)
C2—C3—C4	119.7 (13)	C10—C1—H1	119 (4)
C3—C4—C11	120.8 (10)	C1—C2—H2	119 (2)
C11—C5—C12	118.9 (4)	C3—C2—H2	119 (2)
C7—C6—C12	120.9 (14)	C2—C3—H3	120 (2)
C6—C7—C8	119.4 (15)	C4—C3—H3	120 (2)
C7—C8—C9	122 (2)	C3—C4—H4	119.6 (12)
C8—C9—C13	121.3 (18)	C11—C4—H4	119.6 (11)
N1—C10—C1	118.9 (10)	C11—C5—H5	120.5 (4)
N1—C10—C11	124.5 (8)	C12—C5—H5	120.6 (5)
C1—C10—C11	116.6 (11)	C7—C6—H6	119.5 (15)
C4—C11—C5	122.5 (5)	C12—C6—H6	119.6 (12)
C4—C11—C10	120.0 (7)	C6—C7—H7	120 (3)
C5—C11—C10	117.6 (5)	C8—C7—H7	120 (2)
C5—C12—C6	122.4 (8)	C7—C8—H8	119 (3)
C5—C12—C13	117.7 (6)	C9—C8—H8	120 (3)
C6—C12—C13	119.8 (8)	C8—C9—H9	120 (5)
N1—C13—C9	119.0 (13)	C13—C9—H9	119 (5)
